# Specific health beliefs mediate sex differences in food choice

**DOI:** 10.3389/fnut.2023.1159809

**Published:** 2023-06-05

**Authors:** Viktoria S. Egele, Robin Stark

**Affiliations:** Department of Educational Research, Saarland University, Saarbrücken, Germany

**Keywords:** food choice, health beliefs, sex differences, mediation, diet

## Abstract

**Objective:**

Although sex differences in dietary habits are well documented, the etiology of those differences is still a focus of research. The present study examines the role of specific health beliefs regarding healthy amounts of food for food choice and its relation to sex, more specifically, the assumption that sex differences in food choices are mediated by differentiating health beliefs.

**Method:**

212 German participants (44.3% female) aged 18–70 answered an online self-report questionnaire on their dietary habits and health beliefs, based on the recommendations of the German Nutrition Society.

**Results:**

Most of the anticipated sex differences in food choice and some differences in health beliefs were found. The mediation hypothesis was partly supported, as the relationship between sex and fruit, vegetable, and fish consumption was mediated by the respective health beliefs. However, no mediation effects were found for meat, egg, cereal, and milk product consumption.

**Conclusion:**

The support for the mediation hypothesis aligns with previous findings and indicates that health beliefs might be an important pathway to fostering healthier food choices, especially for men. Nonetheless, sex differences in food choice were only partially mediated by sex differences in specific health beliefs, indicating that future studies might benefit from parallel mediation analyses to reveal the impact of other relevant factors influencing sex differences in food choice.

## 1. Introduction

Poor diet is one of the most important issues nowadays, both in terms of a burden for the healthcare system as well as an influence on individual’s health and overall quality of life (1–3). As the rates of obesity and overweight have nearly doubled since the 1980s, and obesity is mainly attributed to poor diet and eating habits (4, 5), fostering preventive health behavior is of great importance. Although poor diet is a nationwide phenomenon, men seem to be even more affected by it than women, as the prevalence of diseases associated with poor nutrition [e.g., obesity, cardiovascular disease, and diabetes (2) is higher for men (6–8)]. A search for causes has consistently shown that sex differences in dietary patterns are an important influencing factor (9–11). Since food choices are significantly influenced by health beliefs and it is known that men’s and women’s health beliefs differ, it is important to take a closer look at the role of health beliefs regarding dietary behavior depending on sex. A deeper understanding of the etiology of sex-related differences in food choice might allow to tailor interventions more specifically to the target group and, therefore, work more effectively.

A large number of studies demonstrate that men’s diets differ from women’s and can sometimes be seen as worse than women’s (12, 13). Women make food choices that are closer to the national dietary guidelines (14), which is evident in a wide range of food categories. For example, women consume more fruit and vegetables, as well as more high-fiber foods than men, such as cereals and whole-grain bread (15–18). In contrast to this, men eat more eggs, milk products, fish, and meat than women (15, 18, 19), and a larger amount of food in men’s diets is derived from animal products (20). Recent studies also show that women report a lower liking of meat than men (21) and tend to avoid meat in their diet (22). Men report a higher consummation of eggs than women (15). Wham and Worsley (23) showed that sex was a significant predictor of milk consumption, men drank more milk than women, and fewer men than women were non-consumers. Similar findings concerning milk product consumption were also found in longitudinal analyses (24).

Although sex differences in dietary patterns have been demonstrated in many studies, less is known about the etiology of the differences in food consumption. Even though the importance of understanding the influences on food choice for men and women has been recognized for years the topic is still the subject of scientific studies today (21, 25–28).

Food choices are determined by psychological, social, and cultural factors (21, 29–32). However, health beliefs seem to be of particular importance when it comes to explaining sex differences in food choices (9). For example, predisposing factors, such as beliefs, knowledge, and attitudes, and reinforcing factors, such as personal resources, were found to be associated with actual fruit, vegetable, and meat consumption (21, 33, 34). Further, especially the belief in the importance of a high fruit and vegetable diet had a great impact on fruit and vegetable intake. Also, more variance in fruit and vegetable intake was explained by psychological factors than by demographic characteristics.

Individuals’ beliefs about specific foods and eating behavior can be characterized as action-regulation variables (35). Health beliefs can be defined as one’s personal experience about all health-related topics (36). In contrast to knowledge, a belief is not based on objective principles or learned facts but on one’s personal experience (37, 38). Health beliefs and their effects on food choice have been examined previously (39–44), indicating that health beliefs are strongly and positively associated with food choices (32, 45).

Health beliefs differ between men and women (9, 22). Concerning general diet-related health beliefs, women rate the factor “health” as more important than men when making food choices (15, 22). The findings of general diet-related health beliefs are also reflected in health beliefs regarding specific food categories. For example, it has been shown that women consider nutrient-dense foods as well as high-fiber foods like cereals, fruit, and vegetables (15, 29) to be healthier than men. Consistent with these findings, women attribute significantly more importance to health beliefs such as “avoiding high-fat foods,” “adequate intake of fiber,” “adequate consumption of fruit” and “avoiding additional salt” (11). Analogously, men believe high meat consumption to be desirable (46, 47) and consider a diet with meat and fish as more important for their health than women do (48). Men also value eggs more than women (15). Regarding milk products, differences in health beliefs are not entirely clear, as Wham and Worsley (23) stated. Women seem to have more positive attitudes toward them, but they are also more concerned about the fat content (49). It also seems that men and women like milk products equally (15).

As outlined above, sex-related differences in health beliefs and actual food consumption have often been replicated. Furthermore, numerous studies provide evidence for a close relationship between health beliefs about food and actual food consumption (9, 22, 32).

First evidence indicates a mediating function of health beliefs. For example, several studies have shown that women eat more foods that they consider to be beneficial to their health (22) and which are in line with their life goals (50). In addition, Wardle and colleagues (11) tested the relationship between sex, food choice (i.e., fat, high-fiber foods, fruit, and salt), and health beliefs. The particular health beliefs referred to the importance of the food choice for one’s health on a scale from 1 (very low importance) to 10 (very high importance). In this study, they demonstrated that health beliefs are associated with both sex and actual dietary behaviors, and have a mediating effect (11). Recent studies show that about 40% of sex differences in food choices can be explained by health beliefs (9).

Despite these remarkable first results, the effects of specific health beliefs on sex differences in food choices have not been studied extensively and were limited to rather superficial assessments (11). There is a research gap concerning the role of specific health beliefs about healthy amounts of food for food choice and its relation to sex. Kraus (35) showed in a meta-analysis that the correlations of self-reported health behavior with beliefs are higher when the levels of specificity in beliefs and behavior are comparable. Thus, to foster a deeper understanding of the food choices of men and women, it seems obvious to examine the role of very specific health beliefs, namely beliefs on the amount of food that is considered to be healthy.

Therefore, the goals of the present study are as follows: First, previously shown sex differences in food choices ought to be replicated. Secondly, sex differences in specific health beliefs (i.e., food choices personally considered to be healthy) will be examined. Thirdly, the mediating role of these specific health beliefs on sex differences in food choices will be investigated.

*H1*: Women eat more fruit, vegetables, and cereals than men. Men eat more meat, fish, eggs, and milk products than women.

*H2*: Women believe larger amounts of fruit, vegetables, and cereals to be healthy rather than men do. Men believe larger amounts of meat, fish, and eggs to be healthy more than women do. For milk products, sex differences will be examined exploratively.

*H3*: The association between sex and actual food consumption is mediated by specific health beliefs.

## 2. Methods

The conduct of this study complied with the ethical standards of the responsible committee (Anonymized).

### 2.1. Sample

Previously reported effect sizes of sex differences in diet and physical activity were mostly small (29), thus, small effects were anticipated (51). The intended sample size calculated by G*Power 3.1 was 212 participants (52). The acquired sample included 216 German participants. Four participants were excluded from the analyses, as they did not complete the entire questionnaire and dropped out. Therefore, the final sample contained 212 participants (44.3% female) between the ages of 18 and 70 (*M* = 31.03, SD = 13.65). The mean age of the 94 female participants was 25.87 years (SD = 9.82, range 18–60), and the mean age of the 118 male participants was 35.14 years (SD = 14.87, range 18–70). 75% of the sample had graduated from high school and nearly 32% of those participants had a university degree.

### 2.2. Instruments

For demographics, sex, age, and education were assessed by rating scales.

Dietary habits were measured with seven items based on the recommendations of the German Nutrition Society (53). The German Nutrition Society divides food into seven groups: vegetables, fruit, cereals, milk products, meat, fish, and eggs. It is recommended to eat at least three portions of vegetables per day, as well as at least two serving sizes of fruit per day, two serving sizes of cereals, and two serving sizes of dairy products. In addition, it is recommended to consume two serving sizes of meat, two serving sizes of fish, and three eggs per week. To assess dietary habits, the subjects´ average amount of servings consumed per day was assessed for vegetables, fruit, cereals, milk products, meat, fish, and eggs (e.g., How many servings of fruit did you eat on average per day in the last 7 days?). The items translated from German are included in the Supplementary material. Here, to ensure better comparability, the approach of other authors was followed (29), who also suggested working with serving sizes (50). Therefore, before answering the questions, subjects were presented with an example item for a fruit and vegetable serving, which were based on the guidelines of the German Nutrition Society (53). A short-form consumption questionnaire was chosen, as it was shown previously that the short-form achieves similar precision to a long-form questionnaire (i.e., a detailed query of fruit and vegetable types), with the advantage of being quicker to answer (54).

Health beliefs of dietary behavior were assessed similarly to the actual dietary behavior. The number of servings per day that the subjects considered healthy were assessed for vegetables, fruit, cereals, milk products, meat, fish, and eggs (i.e., How many servings of fruit do you consider to be healthy?). Again, the items translated from German are included in the Supplementary material. Dietary habits and health beliefs were assessed using an open-response format to avoid the confounding effects of a forced-choice format (55).

### 2.3. Procedure

All hypotheses were specified before the data were collected. The online questionnaire was implemented using SoSci Survey (56). First of all, participants gave informed consent before taking part and agreed to the data protection regulation. Then, they provided information on their health beliefs. Thereafter, health behavior was assessed. Finally, subjects were asked to provide information about their attitudes and socio-demographic data.

### 2.4. Analytic strategies

Data analysis was conducted using IBM SPSS Statistics 28 and version 3.2 of the PROCESS macro by Hayes (57). The significance level was set at *α* = 0.05. Outliers were excluded based on absolute deviation around the median, as suggested by Leys et al. (58) because this method is considered particularly robust.

As food intake is known to change as individuals age (59), age was included as a covariate in all analyses. Analyses of covariance (ANCOVA) were used to analyze if food consumption and health beliefs differ between men and women, controlling for age. To test whether the relationship between sex and food consumption was mediated by specific health beliefs, the PROCESS macro module was used (57). In this analysis, testing the indirect path is recommended to determine mediating effects (57, 60). The assumptions for the statistical procedures were ensured before the data analyses. Data is available on request due to privacy and ethical restrictions.

## 3. Results

### 3.1. Testing for sex differences in food consumption and respective health beliefs

As displayed in Table 1, women ate significantly more fruit, vegetables, cereals, and eggs, than men, who ate significantly more meat than women. Regarding fish and milk product consumption, no significant sex differences were found. The covariate, age, was not significantly related to fruit, vegetable, cereal, meat, and milk product intake, but there was a significant relation of age to fish intake. In support of hypothesis 1, women reported eating more servings of fruit, vegetables, and cereals per day, and men reported eating more meat. However, contrary to the hypothesis, women reported eating more eggs per day and the expected sex differences for fish and milk products consumption were not found.

**Table 1:** Descriptives and test statistics for Hypothesis 1 (food choice) and Hypothesis 2 (health beliefs).

Measure	Men			Women			Sex differences				Covariate: age			
	*n*	*M*	*SD*	*n*	*M*	*SD*	*F*	*df*	*p*	*η_p_* ^2^	*F*	*df*	*p*	*η_p_* ^2^
Food choice														
Fruit	111	1.31	1.13	86	2.10	1.29	18.46	1, 194	<0.001	0.09	< 0.01	1, 194	0.958	< 0.01
Vegetables	109	1.44	1.26	78	2.79	1.42	34.96	1, 184	<0.001	0.16	3.29	1, 184	0.071	0.02
Meat	114	1.25	1.29	88	0.85	1.09	5.16	1, 199	0.024	0.03	0.02	1, 199	0.889	< 0.01
Fish	83	0.09	0.12	63	0.02	0.07	2.93	1, 143	0.089	0.02	15.25	1, 143	< 0.001	0.10
Eggs	109	0.46	0.53	83	0.70	0.77	6.34	1, 189	0.013	0.03	0.27	1, 189	0.605	< 0.01
Cereals	108	1.74	1.36	81	2.47	1.56	8.90	1, 186	0.003	0.05	0.31	1, 186	0.581	< 0.01
Milk products	109	1.19	1.02	82	1.13	0.86	0.16	1, 188	0.687	< 0.01	< 0.01	1, 188	0.965	< 0.01
Health beliefs														
Fruit	116	1.64	0.98	87	2.30	0.99	25.14	1, 200	<0.001	0.11	2.17	1, 200	0.143	0.01
Vegetables	115	1.82	1.25	88	2.95	1.22	27.40	1, 200	<0.001	0.12	10.96	1, 200	0.001	0.05
Meat	117	0.59	0.49	92	0.50	0.57	1.63	1, 206	0.203	0.01	0.49	1, 206	0.484	< 0.01
Fish	117	0.48	0.39	91	0.69	0.54	9.89	1, 205	0.002	0.05	0.03	1, 205	0.859	< 0.01
Eggs	115	0.65	0.57	89	0.73	0.61	0.28	1, 201	0.599	< 0.01	1.78	1, 201	0.183	0.01
Cereals	111	1.27	0.80	82	1.60	0.85	2.96	1, 190	0.087	0.02	6.37	1, 190	0.012	0.03
Milk products	118	1.18	1.08	94	1.23	1.30	0.62	1, 209	0.432	< 0.01	2.09	1, 209	0.150	0.01

Food consumption and health beliefs are scaled in portions per day. *n*, sample size; *M*, mean; *SD*, standard deviation; *F*, *F*-statistic; *p*, value of *p*; *df*, degrees of freedom; *η_p_*^2^, partial eta-squared.

Moreover, women considered significantly larger amounts of fruit, vegetables, and fish, to be healthy, than men did, whereas no significant sex differences in health beliefs were found for meat, milk products, eggs, and cereals. The covariate, age, was significantly related to beliefs on vegetables, and cereals, but not to beliefs on fruit, meat, fish, eggs, and milk products. Results supported hypothesis 2 for health beliefs on fruit, and vegetables, but not for the remaining food categories, where differences were either non-significant (meat and cereals) or contrary to the hypothesis (fish). Beliefs about milk products did not differ for men and women.

### 3.2. Testing for mediating effects of health beliefs on the relationship between sex and food consumption

**Figure 1:**
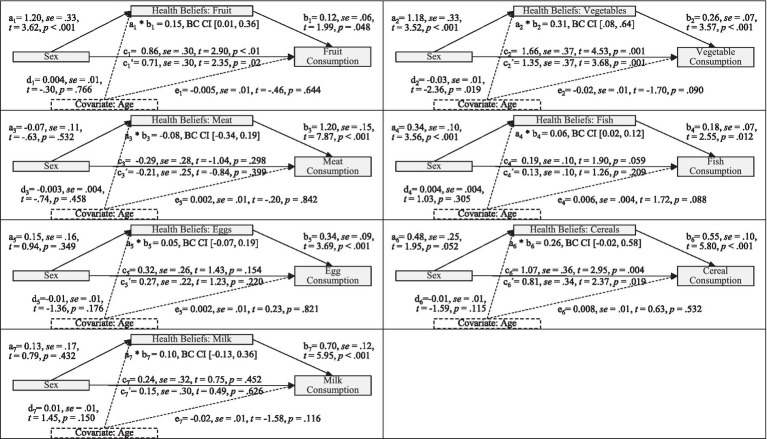
Statistical models for a mediating effect of health beliefs on the relation of sex and food choice (Hypothesis 3). a: effect of gender on health beliefs (women are coded 1, men 0); b: effect of health beliefs on consumption; c: total effect of sex on consumption; c’: direct effect of sex on consumption; d: effect of age on health beliefs; e: effect of age on consumption; a*b: indirect effect of sex on consumption mediated by health beliefs while controlling for age; t: test statistic; *se*: standard error; BC CI: 95% bias-corrected bootstrap confidence interval.

The PROCESS macro module by Hayes (57) was used to test if health beliefs mediate the relationship between sex and food consumption. In these analyses, sex was included as the independent variable, food consumption as the dependent variable, and health belief as a mediator. As a covariate, age was included in the analyses. For each food category, a separate mediation analysis was conducted. As depicted in Figure 1, health beliefs fully mediated the effects of sex on fish consumption. Sex differences in fruit consumption as well as in vegetable consumption were partially mediated by specific health beliefs. Sex differences in meat, egg, cereal, and milk product consumption were not mediated by specific health beliefs. Thus, hypothesis 3 was partly supported.

A concise overview of the hypotheses and results is presented in Table 2.

**Table 2:** Overview of the fingings for Hypothesis 1 (food choice), Hypothesis 2 (health beliefs), and Hypothesis 3 (mediation).

	Food Choice		Health Beliefs		Mediation	
Measure	Hypothesis	Finding	Hypothesis	Finding	Hypothesis	Finding
Fruit	Women eat more fruit than men.	✓	Women believe larger amounts of fruit to be healthy.	✓	The association between sex and fruit consumption is mediated by specific health beliefs.	✓
Vegetables	Women eat more vegetables.	✓	Women believe larger amounts of vegetables to be healthy.	✓	The association between sex and vegetable consumption is mediated by specific health beliefs.	✓
Meat	Men eat more meat.	✓	Men believe larger amounts of meat to be healthy.	✓	The association between sex and meat consumption is mediated by specific health beliefs.	▬
Fish	Men eat more fish.	✓	Men believe larger amounts of fish to be healthy.	✘	The association between sex and fish consumption is mediated by specific health beliefs.	✓
Eggs	Men eat more eggs.	✓	Men believe larger amounts of eggs to be healthy.	▬	The association between sex and egg consumption is mediated by specific health beliefs.	▬
Cereals	Women eat more cereals.	✘	Women believe larger amounts of cereals to be healthy.	▬	The association between sex and cereal consumption is mediated by specific health beliefs.	▬
Milk products	Men eat more milk products.	▬	Exploratory analysis.	▬	The association between sex and milk product consumption is mediated by specific health beliefs.	▬

✓, expected differences; ▬, no significant differences; ✘, significant differences contrary to hypothesis.

## 4. Discussion

The current study investigated the effects of specific health beliefs on the relation between sex and health behavior. Firstly, a replication of previously shown sex differences in food choices was attempted. Secondly, sex differences in specific health beliefs (i.e., food choices personally considered to be healthy) were examined. Thirdly, the mediating role of these specific health beliefs on sex differences in food choices was investigated.

### 4.1. Sex differences in health behavior and health beliefs

In line with previous findings, it was replicated that women eat more fruit, vegetables, and cereals than men, while men eat more meat. Health beliefs for fruit and vegetable consumption were also replicated. This corresponds with previous studies reporting that women consume more fruit and vegetables (15, 17, 29), just like the findings that women consider fruit and vegetables to be healthier than men and attribute significantly more importance to an adequate consumption of fruit (11, 15, 29).

Whereas the finding that women report consuming larger amounts of cereals is compatible with previous findings (16, 18), differences in health beliefs on cereals were not found in the present study. A similar pattern appeared for meat. As expected, men reported eating more meat than women. Although previous findings highlight the importance of meat for men (46, 47), no sex differences in the health beliefs on meat were found yet. Perhaps, women accord higher importance to cereals and men attribute higher importance to meat, but the amount of cereals resp. meat considered healthy does not vary for men and women.

Contrary to previous findings, women reported eating more eggs than men and no significant sex differences were found in the health beliefs concerning eggs. Hu and colleagues (61) discuss the possibility of inaccurate self-reports concerning egg consumption. Although previous studies have shown that egg consumption can be reported with relatively high accuracy (62, 63), it is unclear whether that was the case in the present study and whether the accuracy is comparable for men and women. As women tend to have higher knowledge of nutrition and diet (64), it might be possible that they are better at assessing which foods contained eggs, which might explain the higher reported consummation and the missing differences in the health beliefs.

In the present study, no significant sex differences were found in the reported fish consumption, which contradicts the assumption that men eat more fish than women which has been shown specifically for wild fish (65) as well as raw oysters (66). The sex differences in health beliefs on fish seem to contradict previous literature, claiming that men believe a diet with fish to be more important than women (48). But, as fish consumption correlates positively with the consumption of other foods that are considered healthy [e.g., fruit and vegetables (67)], and women, in particular, eat more “healthy foods” (17, 68), perhaps this phenomenon resulted in the missing differences in the reported fish consumption and explains the differences in health beliefs. Furthermore, some previous studies found similar patterns with women considering seafood and grilled fish slightly healthier than men, descriptively, but did not differ significantly in their beliefs (69).

Regarding milk product consumption, the anticipated differences in behavior were not found, and there were also no significant sex differences in the respective health beliefs. Perhaps, the ambivalence as stated by Wham and Worsley (23) with women being more appreciative of the nutritional value than men, but concerned about the fat content, eclipses potential sex differences. In addition to this, there has been an increasing controversy regarding milk products in recent years (70). For example, there have been claims that dairy products increase the risk of chronic diseases like obesity, type 2 diabetes, cardiovascular diseases, osteoporosis, and cancer. Therefore, it seems as if milk products have become less relevant to people’s lifestyles, and there is increasing skepticism about the health consequences of consuming milk products (23). Yet, amongst those who consume milk products, the health benefits of these products are still valued highly both by men and women (63).

In previous studies, the effect sizes for the sex differences in health behavior and health beliefs were rather small (11, 29). In this study, sex differences in food consumption and health beliefs were more articulated with medium to high effect sizes (51, 68). An evident explanation comes from the statistical analyses used. As potential age-related differences in food intake could have impacted not only food intake (59), but also health beliefs and the relation of both, age was included in all analyses as a covariate. The rationale for this decision is not only supported theoretically but seems to be particularly relevant in this study because of systematic age differences between the two quasi-experimental groups despite the random selection of participants. Not including age as a covariate, in this case, might have led to confounding gender effects with age effects. From an empirical standpoint, however, controlling potential age effects did not seem to be quite as relevant; the results of the statistical analyses and the effect sizes changed only marginally, when not controlling age effects in food choice. Therefore, contextual explanations are also drawn upon. Perhaps, the more pronounced sex differences in food choice can be explained by cultural factors, as the importance of cultural heritage for food choice and food preferences has previously been established (71). Future studies could consider cultural factors regarding dietary behavior in addition to the influence of health beliefs.

Considering these reflections, the results of the present study are rather alarming in terms of external validity, since considerable sex differences in health beliefs and food choices were found in a rather educated German sample. The sample consisted mostly of well-educated participants. In the present sample, nearly 75% of participants had graduated from high school. Compared to nationwide data regarding educational attainment, the sample, therefore, does not seem representative of Germany, where an average of 32% of adults have a high school diploma (72). In the present study, the higher percentage of participants with higher educational attainment may have led to a variance restriction in health behaviors, presumably making the effects more pronounced in a more heterogeneous sample. It seems possible that sex differences in food choices and health beliefs might be even more articulated in a sample with a wider range of education and socioeconomic status (11). Correlations of health behaviors, such as healthy eating and intelligence as well as socioeconomic status, have been widely documented (73, 74). Multiple studies yielded evidence for a positive relation between the adoption and adherence to healthy eating practices and better health outcomes (75, 76), indicating that the articulated sex differences in food choice and health beliefs might translate into nutritional differences and impact health in the long term (11). Thus, the need for research to understand sex differences and their provenance in dietary intake and health beliefs seems even more important.

### 4.2. The mediating effect of specific health beliefs

As expected, health beliefs mediated the relation between sex and actual food consumption for fruit, vegetables, and fish. Analogous to the findings of Wardle and colleagues (11), sex differences in food choices concerning fruit and vegetables were partially mediated by sex differences in specific health beliefs. Thus, differences in health beliefs could explain sex differences in food choice, but even after the introduction of the mediator, the predictor still has some remaining effects. Wardle and colleagues (11) hypothesized that women might be more concerned about health considerations and also more likely to translate those attitudes into actions, which might explain the remaining sex differences in food choices.

Sex differences in fish consumption were fully mediated by health beliefs. Perhaps, many other influencing factors that play an important role in fruit and vegetable consumption, like the “5 a day” campaigns (77, 78), are less important for fish consumption. Therefore, factors like health beliefs may explain most sex differences in fish consumption. It is also possible that this finding is more like a testing effect: if participants do not exactly know what they believe to be a healthy amount of fish to eat per day, they might simply be basing their beliefs on their behavior. This assumption would lead to higher correlations between behavior and beliefs. Nonetheless, in this study, the correlation between the health beliefs for fish and fish consumption was not higher than the respective correlations for the other six food categories.

Statistics failed to demonstrate evidence of sex differences in food consumption being mediated by specific health beliefs for meat, eggs, cereals, and milk products consumption. Several explanatory approaches can be used to interpret these results. It is conceivable that there might actually be no mediating effect in this respect, since the test power should have been given by the sample planning preceding the data collection (described in the methods section).

However, it is also possible that the questioning of health beliefs was too coarse, i.e., one should not have asked for the categories given by the German Nutrition Society [e.g., “cereals” (53)], but with higher specificity (e.g., white flour products, whole grain products, etc.). Indeed, previous research demonstrated gender differences at this level of specificity (79). However, this is contradicted by the fact that gender differences in consumption were mostly found when considering food choice. Therefore, the health beliefs were perhaps covered too specifically to take a mediating role. Previous studies have often assessed broader health beliefs and focused on fewer food categories (11). Concerning the food categories, which did not provide clear mediation effects in the present study, unfortunately, few previous findings have been published so far so replication is required at this point. A conceptual replication could consider whether more specific health beliefs or broader health beliefs than in the present study take on a mediating role with respect to the relationship between gender differences and food choice.

### 4.3. Implications for research and practice

Our findings suggest that specific health beliefs explain some of the sex differences in dietary behavior. Given the rather suboptimal health behavior, especially among men, it seems appropriate to tailor different health interventions, to adapt the health education for men and women, and to target primarily the modification of specific health beliefs of men in the future. Similar conclusions were drawn by recent studies (21). Although an intervention targeting knowledge on healthy eating might not be sufficient to elicit behavioral consequences, since health beliefs may be influenced by knowledge but they are more based on personal experience than on objective principles or learned information (37, 38), numerous previous studies found significant gaps in knowledge about the basic recommendations for a healthy diet. On top of that, there were considerable sex differences in knowledge, and men had even less knowledge than women (64). Thus, interventions fostering knowledge on healthy eating, for example regarding the benefits of a plant-based diet rich in fruits and vegetables, could result in men changing their specific health beliefs to be closer to the official recommendations for healthy behaviors.

However, as mentioned before, according to the mediation results, other factors influencing food choice may also play an important role in sex differences in healthy eating, like food preferences or attitudes toward health. For example, sex differences in the beliefs of the importance of healthy eating were shown to contribute to the sex differences in food choices, as well as differences in dieting beliefs (11). For future research, examining the different influencing factors by parallel mediation analyses seems to be relevant, as it seems plausible that both general health beliefs, whether a food is more “healthy” or “unhealthy,” in combination with specific health beliefs on the optimal amount of food, as well as general dieting beliefs, may serve as mediators for the relation of sex and health behavior. Furthermore, extending the research on the role of health beliefs to other food categories than fruit and vegetables, as demonstrated in this study, seems to be of great importance to address existing research gaps and interpretive challenges in this regard.

## 5. Limitations

It must be noted that a limitation of the present work is the survey method. Dietary behaviors were collected retrospectively, using an online self-report questionnaire. This assessment method may have resulted in recall biases, subjects not being able to participate in the study due to the online survey format (80), or socially desirable response behavior (81). Despite these limitations, the assessment method was chosen because it is cost-effective (54) and has a high test economy (82). Nevertheless, the limitations of retrospective self-reports as a means to assess dietary behaviors can be addressed in future studies through situational surveys, third-party interviews, or more objective collection methods.

Another limitation concerns the quasi-experimental research design without measurement repetition. Since there was only one measurement time point, causal interpretations about the relationship between beliefs and behavior are limited (82). However, the study design chosen in the present work offers the advantage that the motivation of the participants can be better maintained, the chosen recording of health behavior is not an intervention compared to diary studies, and the survey in the natural environment is time-saving and is associated with high ecological validity (82).

## 6. Conclusion

This study aimed at exploring the relationship between sex, dietary behaviors, and specific health beliefs. Concerning dietary behavior and specific health beliefs, most previously established sex differences were replicated, for example, the finding that women eat more fruit and vegetables than men, and believe more servings of fruit and vegetables to be healthy. Moreover, sex differences in specific health beliefs were shown to explain sex differences in dietary behavior for fruit, vegetable, and fish consumption. Although other factors influencing food choice and their relation to specific health beliefs should be considered in future studies, the present findings hint at the relevance of tailored health interventions to alter specific health beliefs in men and women to improve their dietary behavior.

## Data availability statement

The raw data supporting the conclusions of this article will be made available by the authors, without undue reservation.

## Ethics statement

The studies involving human participants were reviewed and approved by the Ethics Committee of the Faculty of Empirical Human and Economic Sciences of Saarland University. The patients/participants provided their written informed consent to participate in this study.

## Author contributions

VE: conceptualization, methodology, investigation, data curation, formal analysis, visualization, writing-original draft preparation, writing-reviewing, and editing. RS: supervision, conceptualization, methodology, resources, formal analysis, writing-reviewing, and editing. All authors contributed to the article and approved the submitted version.

## Conflict of interest

The authors declare that the research was conducted in the absence of any commercial or financial relationships that could be construed as a potential conflict of interest.

## Publisher’s note

All claims expressed in this article are solely those of the authors and do not necessarily represent those of their affiliated organizations, or those of the publisher, the editors and the reviewers. Any product that may be evaluated in this article, or claim that may be made by its manufacturer, is not guaranteed or endorsed by the publisher.
