# Supplementary material for: Specific health beliefs mediate sex differences in food choice
Source: Front Nutr. 2023 Jun 5;10:1159809. doi: 10.3389/fnut.2023.1159809 (PMC10277620; doi:10.3389/fnut.2023.1159809)
Supplement: Supplementary file 1 [file Data_Sheet_1.docx]

**Supplementary Material**

Items concerning food intake and specific health beliefs.

**First, there are some questions regarding your actual eating habits.**

How many servings of the following foods did you eat on average per day in the last seven days?

The following portion information serves to better assess your diet and is based on the guidelines of the German Nutrition Society. Comma indications such as "2.5" servings are also possible.

If you have not eaten any of these foods in the last seven days, enter the value "0".

Vegetables (one serving is about the size of your fist)
_______ serving(s)

Fruit (one serving is approximately the size of your fist)

_______ serving(s)

Cereals (one serving corresponds to about 4-5 slices of bread or 50-60 grams of cereals)

_______ serving(s)

Meat (one serving is about 80-100 grams of meat)

_______ serving(s)

Fish (one serving is about 100 grams of fish)

_______ serving(s)

Eggs (one serving is about the size of one egg)

_______ serving(s)

Milk products (one serving is about 200 grams of milk, yogurt, or cottage cheese)

_______ serving(s)

**Now follow questions regarding your understanding of a healthy diet.**

On average, how many servings of the following foods do you consider to be healthy per day?

If you think that the consumption of one of these foods is not necessary for a healthy diet, please indicate the value "0".

The following portion information is provided to help you estimate your food intake and is based on the guidelines of the German Nutrition Society. Comma indications like "2.5" servings are also possible.

Vegetables (one serving is about the size of your fist)
_______ serving(s)

Fruit (one serving is approximately the size of your fist)

_______ serving(s)

Cereals (one serving corresponds to about 4-5 slices of bread or 50-60 grams of cereals)

_______ serving(s)

Meat (one serving is about 80-100 grams of meat)

_______ serving(s)

Fish (one serving is about 100 grams of fish)

_______ serving(s)

Eggs (one serving is about the size of one egg)

_______ serving(s)

Milk products (one serving is about 200 grams of milk, yogurt, or cottage cheese)

_______ serving(s)
